# Comparison Between Mathematical and Software Calculation Methods for the Measurement of the Cross-sectional Area in Upper Airway Imaging

**DOI:** 10.7759/cureus.6106

**Published:** 2019-11-08

**Authors:** Yousef Aljathlany, Kholoud Alamari, Abdullah Aljasser, Abdullah Alhelali, Manal Bukhari, Mohammed Almohizea, Adeena Khan, Ahmed Alammar

**Affiliations:** 1 Otolaryngology, Head and Neck Surgery Department, King Saud University Medical City, Riyadh, SAU; 2 Radiology, King Saud University Medical City, Riyadh, SAU

**Keywords:** computed tomography, mathematical calculation, software calculation, neck, upper airway, cross-sectional area

## Abstract

Objectives

This study aimed to compare the results of a software calculation method (SCM) and the mathematical calculation method (MCM) in measuring the cross-sectional area (CSA) at four different upper airway segments.

Methods

The data from the retrospective chart reviews of patients older than 18 years who had undergone computed tomography (CT) of the neck at our tertiary care center between September 2014 and September 2018 were reviewed. Data of patients who were intubated, tracheostomized, had nasogastric tubes, tumors, craniofacial anomalies, trauma, or any pathology that may affect the normal airway anatomy were excluded. We measured the anteroposterior (APD) and transverse diameter (TD) utilizing the CT software. CSA was calculated using both the mathematical formula (MCM) and software (SCM) at the glottis, proximal subglottis, distal subglottis, and tracheal levels. A paired sample t-test was used to determine the significant difference between SCM and MCM at each level.

Results

The data of 100 patients (59% female) were reviewed. There was a significant difference between the SCM and MCM at all four levels. The mean differences between the SCM and MCM were -33.63 mm^2^, -24.20 mm^2^, 6.04 mm^2^ (p < 0.001) at the glottis, proximal subglottis, and trachea, respectively. The mean difference at the distal subglottis was -4.08 mm^2^ (p = 0.01).

Conclusion

Our study found a significant difference between the SCM and MCM in measuring the CSA of the four airway segments. Theoretically, the SCM is more accurate and precise than MCM in measuring CSA; however, we could not prove the superiority of either method.

## Introduction

Extensive work has been done to study airway dimensions in both the adult and pediatric population. Rigid bronchoscopy is considered the gold standard for evaluating upper airways; however, several studies support computed tomography (CT) with multiplanar reformatting for providing comparable and accurate results [1-4]. Compared to magnetic resonance imaging, plain radiography, cadaveric studies, and autopsies, CT scan was better in measuring and evaluating the airway columns [1, 4-7]. The anteroposterior diameter (APD) and transverse diameter (TD) are easily measured by any CT software. The cross-sectional area (CSA) is calculated mathematically from the APD and TD, assuming that the upper airway segments take an oval configuration [8-9]. The software calculation method (SCM) measures the CSA directly using the inbuilt software by drawing a perimeter manually around the area of interest and counting each pixel inside that area. The latter method has not been used previously for measuring the different upper airway segments. However, it has been used for other parts of the body, such as the lower airways, blood vessels, and muscles [10-14]. SCM is theoretically more accurate and precise compared to the mathematical calculation method (MCM) in measuring CSA, but no studies have directly compared the methods.

We hypothesized that the SCM and MCM must show significant differences while measuring the CSA of upper airway segments. Thus, the aim of this study was to compare the results of both SCM and MCM in the CSA measurement of four different upper airway segments and to evaluate the demographic factors that could potentially affect the outcomes of such a comparison.

## Materials and methods

This retrospective chart review included the CT data of patients older than 18 years of age who had undergone a neck CT scan at our center between September 2014 and September 2018. This study design was approved by the Institutional Review Board of the King Saud University Medical City. Informed consent was waived because of the retrospective study design. The same dataset was used in another study with completely different objectives and analysis.

Sections of the neck CT scan that were 3 mm or less in thickness, showing the glottis, subglottis, and the first tracheal ring were selected. The data of patients who had tumors, craniofacial anomalies, trauma, previous airway surgery, or any pathology that might affect the normal airway anatomy were excluded. The presence of intubation, tracheostomy, or nasogastric tube(s) was also part of the exclusion criteria. The exclusion was based on the review of the patient file and the CT images during the same visit.

**Figure 1:**
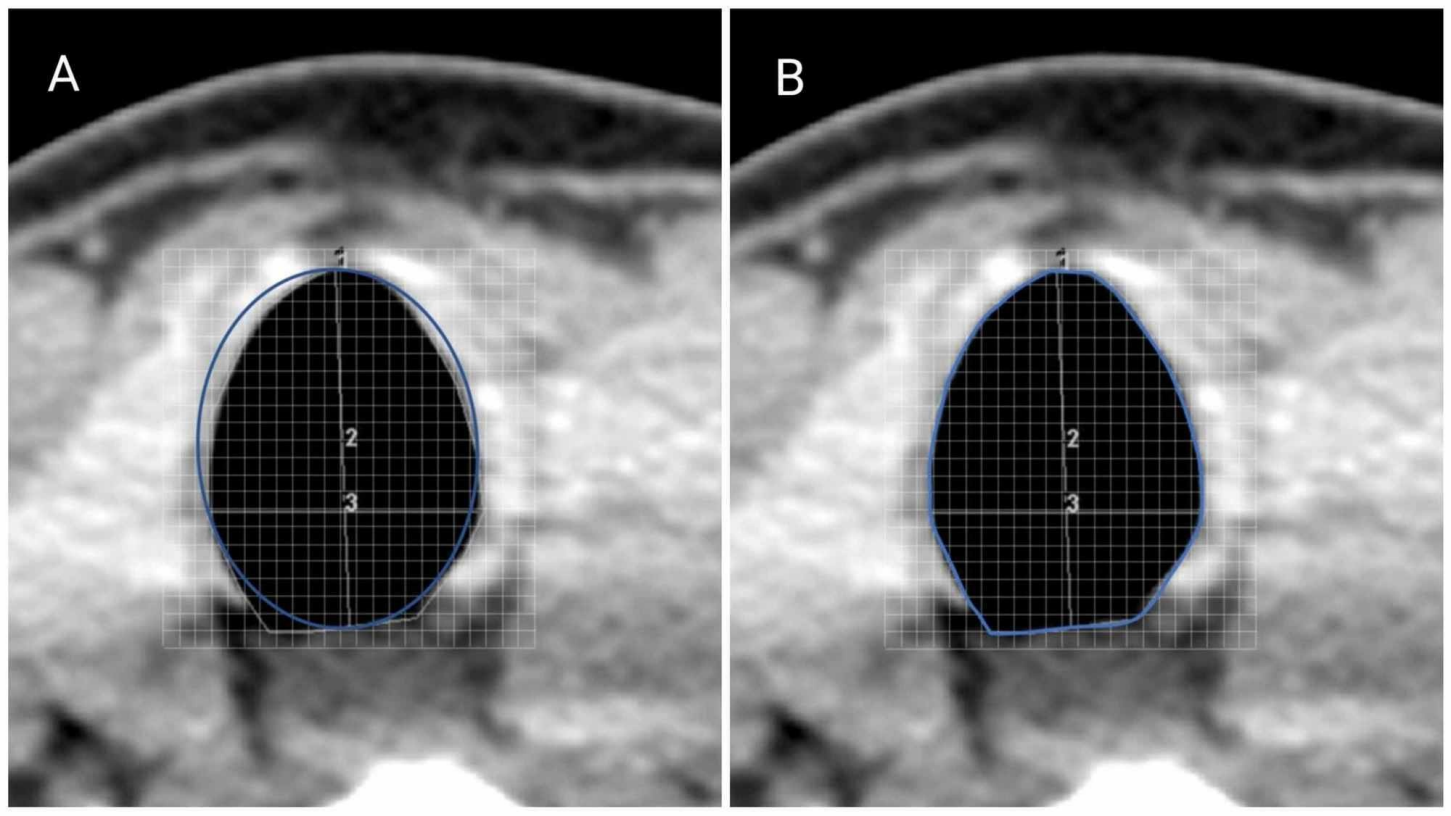
A representative computed tomography (CT) image of the neck comparing the methods of cross-sectional area calculation at the tracheal level. A) An imaginary drawing of the calculated area using the mathematical calculation method is shown in blue; B) The perimeter (blue) was drawn manually using the CT software for directly calculating the cross-sectional area for the software calculation method. 1) The perimeter surrounding the airway column; 2) Anteroposterior diameter; 3) Transverse diameter

A Philips Brilliance iCT 256-slice scanner (Philips Healthcare, Cleveland, OH, USA) was used in all patients. As a routine protocol, the images were obtained during the quiet breathing phase with the patient in the supine position. The images were reconstructed to be parallel to the disc space between the fourth and fifth cervical vertebrae. The slice thicknesses of the scans were 1, 2, or 3 mm. The images were then reviewed using the Centricity PACS RA1000 (GE Healthcare, Barrington, IL, USA) workstation, and the APD, TD, and CSA were measured at the level of the glottis, proximal subglottis (6 mm below the vocal cords), distal subglottis (lower margin of the cricoid), and trachea (first tracheal ring). The APD and TD were manually drawn as straight lines and measured by the software. In MCM, the CSA was calculated using the formula CSA = APD*TD*π*1/4, as shown in Figure 1A. For the software measurement of the CSA, the airway column was manually outlined on the images to form a perimeter, and the total pixels inside that perimeter were calculated where each pixel corresponded to a specific area. The software then computed the CSA by multiplying the total number of pixels by the area of each pixel (Figure 1B) and was directly shown in the software (SCM). The patients’ data, including age, sex, height, weight, and body mass index (BMI), were obtained from their charts on the same day corresponding to the CT scan. 

Statistical methods

To analyze the data in this study, descriptive and inferential statistics were used. The mean and standard deviation were used to describe the results of the CSA using SCM and MCM at each of the four levels; glottic (GL) area, proximal subglottic (PSG6) area, subglottic area at the level of lower cricoid (SGLC), and the first tracheal ring (TR). A paired sample t-test was used to determine the significant difference between SCM and MCM at each level. The level of significance was set at 0.05. A p-value ≤ 0.05 indicated significant differences between the software and mathematical calculations. All statistical analyses were performed using the IBM Statistical Package for Social Sciences (SPSS), version 19 (IBM SPSS Statistics, Armonk, NY, USA).

## Results

The data of 100 patients (male:female = 41:59) were reviewed and categorized by sex, age, height, and BMI. Table 1 shows the demographic profile of the patients. Most patients (31%) were aged 54 - 64 years. In terms of height, 40% measured 154 - 164 cm, followed by 28% who measured 165 - 175 cm. The BMI was 25 - 34.9 in 55% of patients, < 25 in 25% of patients, and ≥ 35 in 20% of patients. When measuring the CSA in mm^2^ using SCM and MCM at the four airway levels, there occurred significant differences between the two methods of measurement at all four levels (Table 2). MCM provided a higher CSA value compared to SCM at all the levels except the TR.

**Table 1:** Distribution of the Demographic Data in the Study

Demographics		%
Sex		
	Male	41
	Female	59
Age (years)		
	19 - 29	18
	30 - 41	22
	42 - 53	20
	54 - 65	31
	66 - 88	9
Height (cm)		
	143 - 153	20
	154 - 164	40
	165 - 175	28
	176 - 192	12
Body Mass Index (kg/m^2^)		
	Underweight (less than 18.5)	3
	Normal Weight (18.5 - 24.9)	22
	Overweight (25 - 29.9)	27
	Obese (30 and above)	48

**Table 2:** Comparison of the Measurement Methods for Cross-sectional Areas at the Four Airway Levels CSA: cross-sectional area; df: degrees of freedom; MCM: mathematical calculation method; SCM: software calculation method; SE: standard error

Level	SCM-CSA Mean (SE)	MCM-CSA Mean (SE)	Mean difference, mm^2^	t-value	df	p-value
Glottis	170.41 (4.91)	204.04 (5.76)	-33.63	-19.35	99	< 0.001
Proximal subglottis	191.99 (5.38)	216.19 (6.73)	-24.20	-11.67	99	< 0.001
Distal subglottis	243.13 (6.6)	247.21 (7.32)	-4.08	-2.61	99	0.0104
Tracheal ring	283.74 (7.59)	277.71 (7.73)	6.04	4.88	99	< 0.001

The comparison of SCM and MCM measurements at the four airway levels were grouped according to the sex, age, height, and BMI, as shown in Tables 3 - 6. SCM and MCM were significantly different at all levels, except at the TR in males and SGLC in females (Table 3). SCM and MCM were comparable in all age groups at the level of the SGLC, and in those aged 42 - 53 years at the level of the TR, while the methods showed statistically different CSA measurements at the remaining levels (Table 4). SCM and MCM measured statistically different CSA at all levels, except at the tracheal level in those whose height was 176 - 192 cm and except at the SGLC level in individuals below 176 cm in height (Table 5). The CSA by both SCM and MCM were not statistically different at all four levels in the underweight group based on BMI. The CSA by SCM and MCM were statistically different in the normal weight and obese groups at all levels, except at the SGLC. In overweight individuals, both methods were statistically different at all levels, except at the level of the TR (Table 6).

**Table 3:** Comparison of the Measurement Methods by Sex GL: glottis; MCM: mathematical calculation method; PSG6: proximal subglottis (6 mm below the vocal cords); SCM: software calculation method; SD: standard deviation; SGLC: distal subglottis (lower margin of the cricoid); TR: tracheal ring

Level	Calculation method	Sex-based cross-sectional area, mm^2^			
		Male		Female	
		Mean (SD)	p-value	Mean (SD)	p-value
GL	SCM	208.5 (46.1)	< 0.001	143.9 (30.2)	< 0.001
	MCM	245.9 (57.7)		174.9 (35.5)	
PSG6	SCM	238.8 (46.5)	< 0.001	159.5 (28.7)	< 0.001
	MCM	275 (60.1)		175.3 (33.3)	
SGLC	SCM	304.7 (47.6)	0.012	200.4 (36.6)	0.430
	MCM	313.2 (62.3)		201.4 (35.6)	
TR	SCM	355.1 (52.3)	0.080	234.1 (42.8)	< 0.001
	MCM	350.8 (56.3)		226.9 (40.3)	

**Table 4:** Comparison of the Measurement Methods by Age GL: glottis; MCM: mathematical calculation method; PSG6: proximal subglottis (6 mm below the vocal cords); SCM: software calculation method; SD: standard deviation; SGLC: distal subglottis (lower margin of the cricoid); TR: tracheal ring

Level	Calculation method	Age-based cross-sectional area, mm2									
		19 - 29 y		30 - 41 y		42 - 53 y		54 - 65 y		67 - 88 y	
		Mean (SD)	p-value	Mean (SD)	p-value	Mean (SD)	p-value	Mean (SD)	p-value	Mean (SD)	p-value
GL	SCM	177.5 (46.6)	<0.001	181.1 (54.2)	<0.001	172.9 (53.9)	<0.001	159.2 (38.1)	<0.001	163.3 (64.5)	0.003
	MCM	210.1 (58.9)		214.1 (66.5)		208.6 (65.3)		194.8 (41.8)		188.9 (66.1)	
PSG6	SCM	190.5 (43.4)	<0.001	199.3 (57.1)	<0.001	194.6 (64.8)	<0.001	186.4 (49.5)	<0.001	190.8 (61.3)	0.012
	MCM	212.6 (55.2)		224.9 (78.3)		220.4 (74.2)		210 (61.9)		213.9 (75.2)	
SGLC	SCM	238.1 (54.7)	0.908	244.7 (61.9)	0.152	239.3 (75.6)	0.122	247.5 (72.2)	0.061	242.9 (64.6)	0.703
	MCM	237.7 (57.8)		250.7 (72.7)		246.3 (88.4)		251.6 (75.2)		244.7 (72.7)	
TR	SCM	279.4 (64.5)	0.021	282.9 (66)	0.012	277.4 (81.2)	0.547	286.7 (85.4)	0.007	298.3 (86.9)	0.005
	MCM	275.4 (63.9)		275 (70.7)		275.5 (86.5)		280 (85.4)		285.9 (83)	

**Table 5:** Comparison of the Measurement Methods by Height GL: glottis; MCM: mathematical calculation method; PSG6: proximal subglottis (6 mm below the vocal cords); SCM: software calculation method; SD: standard deviation; SGLC: distal subglottis (lower margin of the cricoid); TR: tracheal ring

Level	Calculation method	Height-based cross-sectional area, mm^2^							
		143 - 153 cm		154 - 164 cm		165 - 175 cm		176 - 192 cm	
		Mean (SD)	p-value	Mean (SD)	p-value	Mean (SD)	p-value	Mean (SD)	p-value
GL	SCM	130.3 (28.2)	< 0.001	152.4 (30.8)	< 0.001	200.7 (38.2)	< 0.001	226.7 (58.7)	0.002
	MCM	160.3 (32.8)		182.8 (35.4)		241.1 (42.8)		261.2 (82.9)	
PSG6	SCM	151.2 (24.8)	< 0.001	168.9 (35.6)	< 0.001	223.2 (40.1)	< 0.001	264.3 (57.9)	0.002
	MCM	167.4 (34.9)		187.6 (41.1)		254.3 (49.7)		303.9 (85.2)	
SGLC	SCM	192.3 (39.1)	0.372	217.5 (48.9)	0.213	281.9 (52.7)	0.433	323.1 (58.1)	0.049
	MCM	194.3 (39)		220 (53)		284.1 (58.7)		340 (81)	
TR	SCM	230 (46.4)	< 0.001	250.6 (52.6)	< 0.001	325.8 (62.6)	0.019	385.6 (60.4)	0.955
	MCM	219.8 (39.6)		244.6 (51.1)		320.2 (66.3)		385.2 (61.5)	

**Table 6:** Comparison of the Measurement Methods by Body Mass Index (BMI) GL: glottis; MCM: mathematical calculation method; PSG6: proximal subglottis (6 mm below the vocal cords); SCM: software calculation method; SD: standard deviation; SGLC: distal subglottis (lower margin of the cricoid); TR: tracheal ring

Level	Calculation Method	BMI-based Cross-sectional Area, mm^2^							
		Underweight (BMI < 18.5)		Normal Weight (BMI = 18.5 - 24.9)		Overweight (BMI = 25 - 29.9)		Obese (BMI ≥ 30)	
		Mean (SD)	p-value	Mean (SD)	p-value	Mean (SD)	p-value	Mean (SD)	p-value
GL	SCM	163 (28.2)	0.136	182.2 (47.3)	< 0.001	170.2 (50.7)	< 0.001	165.6 (50.3)	< 0.001
	MCM	195.2 (40.4)		212.8 (52.3)		202.6 (52.9)		201.4 (64)	
SG6	SCM	192.7 (17.6)	0.120	202.5 (46.2)	< 0.001	192.1 (55.5)	< 0.001	187 (57.8)	< 0.001
	MCM	226.1 (37.1)		223.2 (54)		213.7 (67.8)		213.7 (74.8)	
SGLC	SCM	265 (29.2)	0.369	257.2 (55.7)	0.406	241.5 (69.8)	0.008	236.2 (69.9)	0.070
	MCM	262.7 (26.7)		255.5 (55.1)		249.7 (76.9)		241.1 (80.9)	
TR	SCM	326.7 (44.1)	0.058	300.8 (66.6)	< 0.001	289.7 (84.9)	0.115	269.9 (74.9)	0.003
	MCM	308.5 (39.5)		293.6 (66.5)		285.8 (82.6)		263.9 (79.8)	

## Discussion

Multiple studies have utilized CT scans to evaluate the upper airway anatomy and its relevance to the demographic factors; however, these studies used MCM for CSA measurements [8-9, 15]. Since MCM assumes that the airways take a regular oval shape, we questioned the accuracy of such measurements based on the actual irregularity of the upper airway segments, the semi-triangular shape of the glottic and proximal subglottic areas, and the indentation of the posterior wall of the trachea with inspiration. Based on the above facts, MCM could miss or underestimate certain areas while overestimating the CSA of other areas. This study confirms our hypothesis that the upper airway CSA at different levels is significantly different when measured by SCM vs. MCM, probably due to the irregular border shapes.

The CSA measurement has the potential of predicting the appropriate endotracheal tube sizing in adults and the possibility of measuring the subglottic or tracheal stenoses severity on CT scans. Also, the study findings could have implications in the design of future studies.

The CSA at the SGLC was significantly different between the two methods, but this difference disappeared on subgroup analyses in females, all age groups, all height groups (except 176 - 192 cm), and in those with BMI ≤ 24.9 (underweight and normal). The similarity between the SCM and MCM calculations can be explained by the presence of the only complete cartilaginous ring of the airway at the level of the cricoid that may not be affected by respiration as compared to the tracheal rings. Also, the lower cricoid segment shows more regularity as compared to the proximal subglottis, explaining the lower magnitude of significance seen at the former level.

The CSA at the level of the GL and PSG6 were consistently different by SCM vs. MCM in all subgroups (except in those with a BMI < 18.5). Thus, relying on MCM to calculate CSA at these levels could cause a strong bias. The underweight group showed no difference between both methods at all levels; however, this could be attributed to the small sample size (n = 3) of this group. Tracheal level measurements showed widely inconsistent results on subgroup analyses. Our explanation is that even though the tracheal level takes a slightly more regular shape, the posterior indentation caused by inspiration could lead to variations as evidenced by our results.

There are some limitations to this study worth mentioning. This study is retrospective in nature. Also, the breathing pattern could affect the airway shape, particularly at the glottic and tracheal levels. This limitation was overcome, in part, by using the same scan for both methods. The sample of some groups may be too small to show significance, and hence the subgroup analysis must be interpreted with caution. Another limitation is the lack of standardization of the CSA measurement as guidance to compare our results, but we can assure that a significant difference between the two methods was shown in all the upper airway segments.

## Conclusions

This study confirms a significant difference between the software-based and mathematical measures of CSA at four levels of the upper airway segments. Theoretically, the SCM is more accurate and precise than the MCM in measuring CSA; however, we cannot prove the superiority of either method based on the results of this study. Future large-sample, comparative studies of CSA employing cadavers and both methods could confirm our findings.
